# Lep gene and leptin concentration in serum of schizophrenia patients with metabolic syndrome

**DOI:** 10.1192/j.eurpsy.2021.438

**Published:** 2021-08-13

**Authors:** A. Boiko, I. Pozhidaev, D. Paderina, E. Kornetova, S. Ivanova

**Affiliations:** 1 Laboratory Of Molecular Genetics And Biochemistry, Mental Health Research Institute, Tomsk National Research Medical Center of the Russian Academy of Sciences, Tomsk, Russian Federation; 2 Department Of Endogenous Disorders, Mental Health Research Institute, Tomsk National Research Medical Center, Russian Academy of Sciences, Tomsk, Russian Federation

**Keywords:** schizophrénia, gene polymorphisms, Metabolic syndrome, leptin

## Abstract

**Introduction:**

Schizophrenia is associated with lower life expectancy due to cardiovascular disease. Metabolic syndrome (MetS) occupies an important place among the main problems. Indicators of hormones regulating metabolism may be appealing candidates as biomarkers of metabolic side-effects. Certain role belongs to genetic factors that might be the basis of sensitivity to development of MetS.

**Objectives:**

The aim is to study polymorphisms of leptin gene (LEP) and serum leptin concentration in schizophrenia patients with metabolic syndrome.

**Methods:**

After obtaining informed consent, patients with schizophrenia (ICD-10: F20) were included: 91 patients for biochemical research and 463 patients for genotyping. Patients were divided into two groups: 46 (119) with MetS; 45 (344) without it. Concentration of leptin was measured on an analyzer MAGPIX (Luminex, USA). Determination of 4 polymorphisms (rs2167270, rs3828942, rs10954173, rs4731426) of LEP was performed by PCR. Differences were considered significant at p<0.05.

**Results:**

The leptin concentration is significantly (p<0.001) higher in MetS (13511.5 [7392.5; 28278.75] pg/ml) compared to patients without MetS (6662 [2131.5; 11380] pg/ml). Significant differences were found in the distribution of rs3828942 (GG:GA:AA): 25.9%:44%:30.2% in MetS and 31.2%:52.6%:16.2% without MetS (χ^2^=10.545, p=0.005). The genotype AA and the allele A have a predisposing effect on the development of MetS (OR_1_=2.247, C.I:1.248-4.046; OR_2_=1.475, C.I:1.093-1.991, χ^2^=6.49, p=0.01).

**Conclusions:**

A number of features are observed in patients with MetS, which impair the functioning of patients. These investigations should aim to optimize the approach to assess the risk of MetS. The study was supported by grants from the RSF 19-75-10012 (genetic research) and 18-15-00011 (determination of leptin concentration)

**Disclosure:**

The study was supported by grants from the Russian Science Foundation №19-75-10012 (genetic research) and №18-15-00011 (determination of leptin concentration)

